# Enhancing Post-Training Muscle Recovery and Strength in Paralympic Powerlifting Athletes with Cold-Water Immersion, a Cross-Sectional Study

**DOI:** 10.3390/ijerph22010122

**Published:** 2025-01-18

**Authors:** Felipe J. Aidar, Wélia Yasmin Horacio dos Santos, Saulo da Cunha Machado, Albená Nunes-Silva, Érica Leandro Marciano Vieira, Diego Ignácio Valenzuela Pérez, Esteban Aedo-Muñoz, Ciro José Brito, Pantelis T. Nikolaidis

**Affiliations:** 1 Graduate Program in Movement Science, Federal University of Sergipe (UFS), São Cristovão 49107-230, SE, Brazil; fjaidar@gmail.com (F.J.A.); weliaa@hotmail.com (W.Y.H.d.S.); saulo0407@gmail.com (S.d.C.M.); 2 Group of Studies and Research of Performance, Sport, Health and Paralympic Sports—GEPEPS, The Federal University of Sergipe—UFS, São Cristovão 49107-230, SE, Brazil; 3 Graduate Program in Physiological Science, Federal University of Sergipe (UFS), São Cristovão 49107-230, SE, Brazil; 4 Inflammation and Exercise Immunology Laboratory (LABIIEX), Federal University of Ouro Preto Sports Center, Ouro Preto 35400-000, MG, Brazil; albenanunes@hotmail.com; 5 Laboratório de Imunofarmacologia, Departamento de Bioquímica e Imunologia (ICB/UFMG), Federal University of Minas Gerais, Belo Horizonte 31270-901, MG, Brazil; ericalmvieira@gmail.com; 6 Escuela de Kinesiología, Facultad de Salud, Magister en Ciencias la Actividad Física y Deportes Aplicadas al Entrenamiento Rehabilitación y Reintegro Deportivo, Universidad Santo Tomás, Santiago 4440000, Chile; diegovalenzuela@santotomas.cl; 7 Departamento de Educación Física, Deportes y Recreación, Universidad Metropolitana de Ciencias de la Educación, Santiago 7750332, Chile; esteban.aedo@usach.cl; 8 Unidad de Ciencias Aplicadas al Deporte, Instituto Nacional de Deporte, Santiago 7750332, Chile; 9 Graduate Program of Physical Education, Federal University of Juiz de Fora, Juiz de Fora 36036-900, MG, Brazil; cirojbrito@gmail.com; 10 School of Health and Caring Sciences, University of West Attica, 12241 Athens, Greece

**Keywords:** Paralympic powerlifting, recovery methods, performance, training

## Abstract

(1) Background: The recovery method is important and decisive in the training system. This study aimed to assess the effects of various post-training recovery methods on muscle damage and strength indicators in Paralympic powerlifting athletes. (2) Methods: A crossover study was conducted involving eleven male athletes (25.4 ± 3.3 years, 70.3 ± 12.1 kg). Muscle damage was assessed using blood biochemical markers (cytokines IL-6, IL-10, and TNF-α) and isometric force indicators, including the maximum isometric force (MIF), time to MIF, and Rate of Force Development. The following assessments were performed before, immediately after, and at 24 and 48 h after the recovery protocol: (a) passive recovery (RP) or (b) cold-water immersion (CWI). (3) Results: The main results indicated that maximum isometric force (MIF) significantly improved after 48 h of CWI application (*p* < 0.05; for all comparison). The analysis of biochemical markers did not yield significant differences between the recovery methods at different time points (*p* > 0.05). For IL-6, there were significant differences between CWI before (2.29 ± 1.08, 95% CI 1.57–3.01) and CWI 2 h later (2 h) (4.59 ± 2.96, 95% CI 2.60–6.57; *p* = 0.045), and between CWI 15 min later (15 min) (4.14 ± 2.24, 95% CI 2.63–5.64) and CWI 48 h later (48 h) (2.33 ± 1.25, 95% CI 1.49–3.17; *p* = 0.034). There were differences between CWI 2 h (4.14 ± 2.24, 95% CI 2.63–5.64) and CWI 48 h later (2.33 ± 1.25, 95% CI 1.49–3.17; *p* = 0.035; F = 9.202; η^2^p = 0.479; high effect). (4) Conclusions: CWI significantly improved the post-resistance training muscle damage and strength in Paralympic powerlifting athletes.

## 1. Introduction

Powerlifting is a sport that involves lifting weights in exercises such as bench press, deadlift, and free squat using the heaviest load possible within the proper technique. In the Paralympic modality, powerlifting is limited only to the adapted bench press, where the difference is that the athlete performs the exercise with the upper limbs on the bench [1]. Consequently, training in this sport typically involves heavy loads and high training intensity. High loads tend to cause an increase in muscle micro injuries [2,3], which increases the athlete’s recovery time between training sessions and affects the initially planned volume. This type of impairment is not desired when it comes to achieving high performance [4]. Training tends to promote fatigue, muscle damage, and an increased risk of musculoskeletal injuries, so for an athlete to maintain their training rhythm, aiming to improve performance, it becomes imperative that they recover adequately between training sessions [5]. This context requires that we create post-workout strategies to accelerate the recovery, aiming to keep all planned training [3,6].

Therefore, some methods are used to improve the post-training recovery, including the use of drugs [7], supplements [8], CWI, and dry needling [3]. Among these methods, CWI stands out, although it remains a subject of controversy, particularly in Paralympic powerlifting [3,9,10]. Recently, two meta-analyses showed that CWI may have a moderate effect on reducing muscle damage and pain [11,12]; however, other meta-analyses have indicated that CWI may have a detrimental effect on the athlete [13,14]. Grgic [13] recommends caution when using CWI, where exposure time and temperature must be individualized. In Paralympic powerlifting specifically, this practice has been little tested, mainly due to the difficulty in immersing athletes in water [3]. Studies indicate that CWI can reduce muscle soreness and inflammation, accelerating the recovery process [15]. However, some research suggests that frequent use of CWI after strength training may potentially impair long-term strength and muscle hypertrophy gains [16]. Regarding isometric strength, it has been observed that CWI can help maintain strength levels after intense exercise, possibly due to its ability to attenuate the acute inflammatory response [3]. Furthermore, CWI appears to positively influence the levels of inflammatory cytokines, contributing to a faster and more efficient recovery of athletes after strength training sessions [17].

Given these considerations, the present study aims to assess the effects of different post-training recovery methods on muscle damage in Paralympic powerlifting athletes. In doing so, we hypothesize that CWI is likely to improve post-training muscle recovery and reduce pro-inflammatory blood immune markers while increasing anti-inflammatory ones.

## 2. Materials and Methods

### 2.1. Study Design

**Figure 1:**
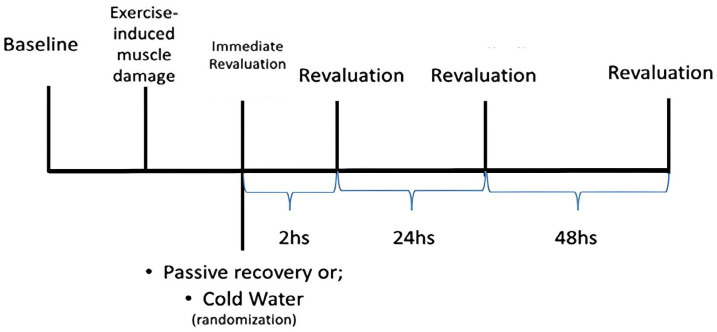
Study design.

This study was conducted over three weeks, with the first week dedicated to familiarizing the subjects with the tests, and the subsequent weeks allocated to the evaluation using different recovery methods: PR and CWI. The choice of recovery method was determined using random selection, by lottery. This was a cross-over study, with half of the sample assigned to each week, until all subjects had completed all the methods, as depicted in Figure 1. In each recovery method (CWI and PR), athletes were assessed before and after recovery method, and 2, 24, and 48 h after training in relation to cytokines. For the evaluation of force indicators, the moments were before and after recovery, and 24 and 48 h later. Participants were instructed not to engage in vigorous physical activity in the previous 24 h and not to change their daily diet. They were also instructed not to use any medications or additional foods, such as coffee or pre-workout supplements. The order of tests during the intervention was always to collect blood initially, and for this type of analysis no type of dietary restriction was necessary. After the blood collection, the athletes performed the force tests.

**Figure 2:**
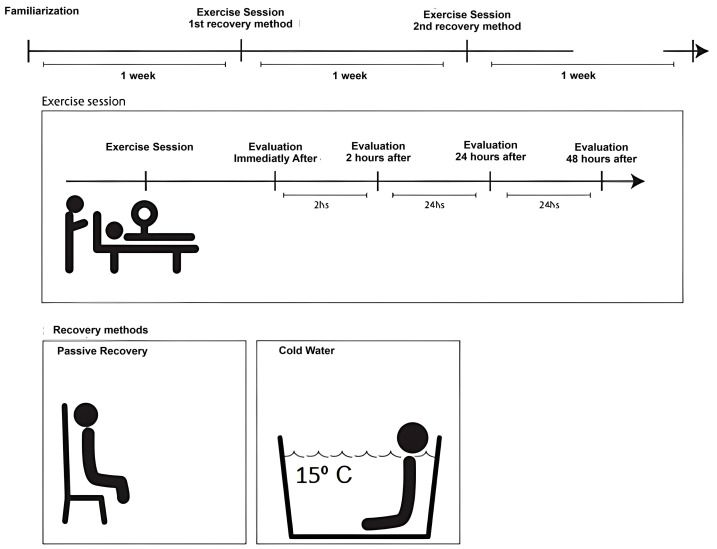
Study schedule.

Figure 2 shows the design of the work, where it can be seen that familiarization was carried out in the first week, while in the second and third weeks the recovery methods were carried out, with 50% doing PR and 50% doing CWI (in the third week this was reversed). The athletes had a minimum rest of 72 h after the intervention. It is worth noting that there was no need to do a wash out, given that studies demonstrate that within 48 h athletes are already recovered [3,18,19,20].

### 2.2. Participants

This was a cross-over study that included 11 Paralympic powerlifting athletes (27.1 ± 4.7 yrs.; 84.6 ± 18.5 kg; 3.2 ± 0.5 yrs. of experience; 150.2 ± 27.5 kg in 1RM bench press; 1.8 ± 0.2 1RM/BW) (Table 1). All subjects were national-level competitors, with some also competing at the international level (all athletes performed ≥1.4 1RM/BW, the limit that classifies them as elite athletes). They were classified as eligible for their respective categories and ranked among the top ten nationally according to the criteria of the International Paralympic Committee [2]. Among the types of disabilities, three athletes had spinal cord injuries due to trauma, with lesions below the eighth thoracic vertebra; two had lower limb malformations (Arthrogryposis); two had sequelae due to poliomyelitis; three had below-knee amputations; and one had cerebral palsy. Among the athletes, there was one who achieved second place in the Americas Open and seventh place in the Paralympics, a second and a third place in the Americas Open, and a junior world 2nd place.

Inclusion criteria included being clinically stable and practicing the sport for at least 18 months, making them eligible for Paralympic powerlifting. Another inclusion criterion was competing officially in the last six months and being ranked among the top 10 in their respective categories at the national level.

The athletes voluntarily participated in the study and signed informed consent forms in accordance with Resolution 466/2012 of the National Commission for Ethics in Research (CONEP) of the National Health Council (CNS), in compliance with the Declaration of Helsinki (1964, revised in 2013) of the World Medical Association. This study was approved by the Research Ethics Committee of the Federal University of Sergipe (ID-CAAE: 67953622.7.0000.5546) under statement number 6.523.247, dated 22 November 2023.

### 2.3. Blood Biochemical Markers (Cytokines)

Plasma cytokine levels in individuals were determined using the Cytometric Bead Array (CBA) method, following the manufacturer’s instructions (Cytometric Bead Array (CBA) Human TH1/TH2 Cytokine Kit II, BD Biosciences, San Diego, USA). We utilized kits for the quantification of inflammatory proteins (cytokines IL-6, anti and pro inflammatory; IL-10, anti-inflammatory; and TNF-α, pro-inflammatory). Plasma samples were briefly incubated with capture microspheres coated with specific antibodies for the respective cytokines and chemokines, as well as the proteins in the standard curve. Subsequently, the color reagent phycoerythrin (PE) was added, and the samples were incubated for 3 h. After incubation, the samples were washed with Wash buffer^®^ and centrifuged (200 rpm, 5 min, room temperature). The supernatant was discarded, and the pellet containing the microspheres was resuspended with 300 µL of Wash buffer. Samples were acquired using the BD FACSCanto II Flow Cytometer (Becton & Dickinson, Franklin Lakes, NJ, USA). The results were analyzed using FCAP software (BD Bioscience, Franklin Lakes, NJ, USA) and are represented in pg/mL.

### 2.4. Isometric Force Indicators

To measure muscle strength, maximum isometric force (MIF), time to MIF (Time), and Rate of Force Development (RTD) were determined using a Chronojump load cell (Chronojump^®^, Bosco System, Barcelona, Spain) secured to a flat bench with Spider HMS Simond carabiners (Chamonix, France), boasting a breaking load of 21 kN, certified for climbing by the Union International des Associations d’Alpinisme (UIAA). A steel chain with a breaking load of 2300 kg was employed to anchor the load cell to the bench [3,22].

**Figure 3:**
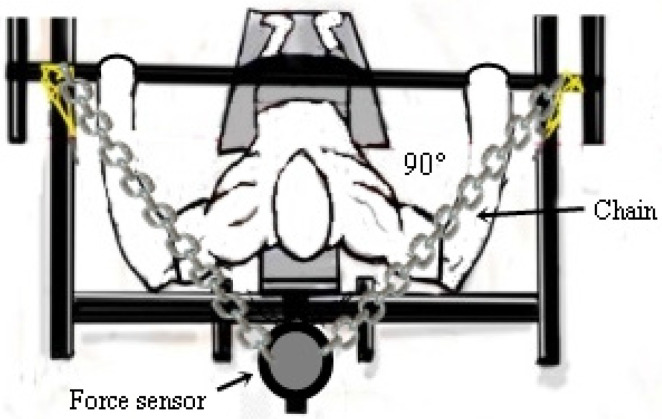
Demonstration of the positioning of the force sensor for evaluation of static force indicators.

The maximum isometric force (MIF) was attached to the adapted bench (Figure 3), adapted from the procedure performed by Fraga et al. [7] and Sampaio et al. [23].

The isometric force was determined using a force sensor attached to an inextensible cable and the adapted bench press. The MIF was measured using the maximum force generated by the muscles of the upper limbs during the bench press exercise. The MIF is determined in Newtons (N) using the formula N = (M) × (C), where M = mass in kg and C = 9.80665, measured between the load cell cable attachment point and the bench press [24]. Participants were instructed to perform a single maximum movement looking for elbow extension (90°) (“as fast as possible”) and to relax. This angle was verified using a device for measuring the range of motion, Model FL6010 (Sanny^®^, São Bernardo do Campo, Brazil). Participants were instructed to perform a single maximal movement to elbow extension (as quickly as possible) and then relax for MIF assessment. Each subject performed three repetitions of 5 s of maximal effort with 10 min of rest between repetitions to measure maximal isometric force. A steel chain was used to fix the load cell to the bench.

The RFD was determined using the force to time ratio until reaching the maximal force (RFD = ΔStrength/ΔTime) [14,16] within 300 ms [24].

### 2.5. Load Determination

For the determination of the maximum load, a One Repetition Maximum (1RM) test was conducted. Athletes selected a weight based on their performance in previous competitions that they could lift only once. The weight was adjusted until the maximum load that could be lifted for a single repetition was reached. If an athlete could not complete a single repetition, 2.4% to 2.5% of the weight used was subtracted [25,26]. Between attempts, a 3–5 min rest period was employed. The 1RM assessment test was conducted at least 72 h prior to the intervention.

### 2.6. Warm-Up (Exercises Pre-Test)

Prior to the intervention (training), athletes underwent a warm-up consisting of shoulder abduction, elbow extension using a pulley, and shoulder rotation. During the warm-up, athletes performed three sets of 10 to 20 repetitions. Following the warm-up, the bench press exercise was performed with 2 sets of 10 repetitions (2 × 10), with a load of 30 to 50% 1RM. Throughout the testing, participants were verbally encouraged to execute the movement forcefully and swiftly [2].

### 2.7. Training Session

The bench press exercise was employed, comprising five sets of five repetitions with a load ranging from 80% to 90% of 1RM. A 3 min rest interval was incorporated between sets [24].

### 2.8. Recovery Intervention

Passive Recovery: A 15 min rest period was provided without any specific protocol, during which the subjects remained seated after the intervention [3,24].

Cold Water Immersion (CWI): Individuals underwent recovery through immersion in a plastic ice-filled pool (11–15 °C ± 0.5 °C) for 15 min [3], in accordance with protocols and studies on the methods [27,28]. During the CWI process, temperature was monitored using the Hikari HTH-240 Digital Thermo-Hygrometer (Hikari^®^, Shenzhen, China) to ensure thermal consistency throughout the procedure. All subjects underwent the recovery method, and the method was performed with the entire body immersed in cold water, with only the head remaining out of the water. All subjects underwent recovery methods, and the method was performed with the entire body immersed in cold water, with only the head remaining above the water. The athletes are elite athletes at national and international level and this method is used in many conditions to support preparation.

### 2.9. Statistical Analysis

For the descriptive analysis, measures of central tendency, mean (X) ± standard deviation (SD) and 95% confidence interval (95% CI), were employed. The normality of the variables was assessed using the Shapiro–Wilk test, taking into consideration the sample size. The data exhibited homogeneity and a normal distribution. Performance comparison between groups was conducted using a Two-way ANOVA test, (Condition and Moment), for repeated measurements, with Bonferroni post hoc analysis. The test to verify homogeneity was the Levene test. Statistical analysis was carried out using the computerized Statistical Package for the Social Sciences (SPSS), version 22.0. The adopted significance level was *p* < 0.05. Effect size was assessed using partial eta squared (η^2^p), with values indicating low effect (≤0.05), moderate effect (0.05 to 0.25), high effect (0.25 to 0.50), and very high effect (>0.50) [29,30].

## 3. Results

Table 2 shows the results for the maximum isometric strength (MIF), time-to-reach MIF, and Rate of Force Development (RFD) before and after the types of recovery.

In terms of the MIF, there were differences between PR Before and 24 h (a), and between 15 min and PR Before (c), and PR 48 h (g). There was a difference between PR Before and PR 24 h (a). There were also differences between the methods at the 24 h time points (e). In time to MIF, there was a difference in the PR condition between 15 min and Before (a), and 48 h (c). There was a difference in PR between Before and 24 h (e). There were also differences between the PR and CWI conditions at the 15 min (d) and at the 24 h (f). In terms of the RFD, there was a difference in PR between the Before and 15 min (a) and 24 h (e). There was a difference between the conditions at the 24 h (f).

**Figure 4:**
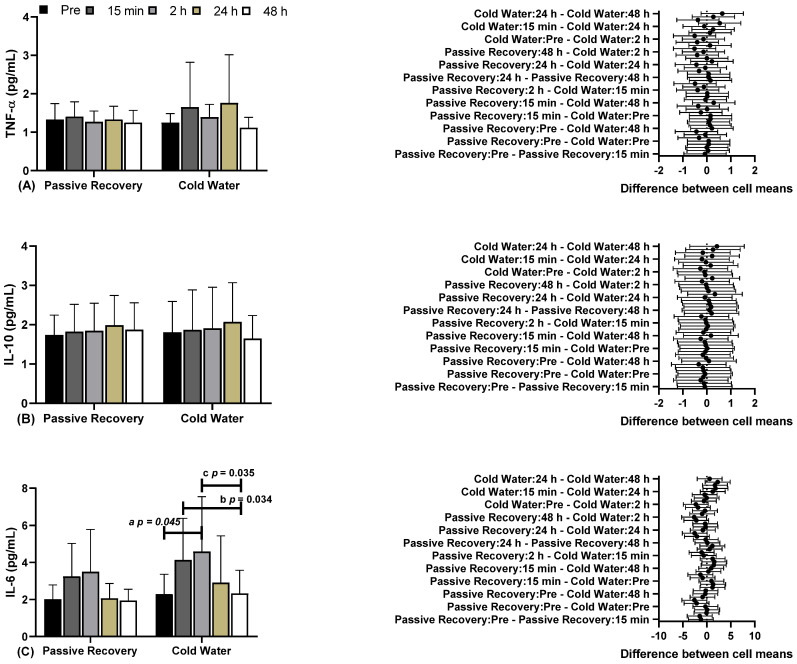
(**A**) Tumor Necrosis Factor Alpha (TNF-α), (**B**) interleukin 10 (IL-10), and (**C**) interleukin 6 (IL-6) in the conditions of passive and cold-water recovery at different time points.

In Figure 4, you can see the results for blood biochemical markers, including Tumor Necrosis Factor Alpha (TNF-α), interleukin 10 (IL-10), and interleukin 6 (IL-6), under passive and CWI recovery conditions at various time points before and after.

Regarding (A) TNF-α and (B) IL-10, there were no differences between time points and conditions. Regarding (C) IL-6, there were significant differences between CWI before (2.29 ± 1.08, 95% CI 1.57–3.01) and CWI 2 h later (2 h) (4.59 ± 2.96, 95% CI 2.60–6.57; “**a**” *p* = 0.045), and between CWI 15 min later (15 min) (4.14 ± 2.24, 95% CI 2.63–5.64) and CWI 48 h later (48 h) (2.33 ± 1.25, 95% CI 1.49–3.17; “**b**” *p* = 0.034). There were differences between CWI 2 h (4.14 ± 2.24, 95% CI 2.63–5.64) and CWI 48 h later (2.33 ± 1.25, 95% CI 1.49–3.17; “**c**” *p* = 0.035; F = 9.202; η^2^p = 0.479; high effect).

## 4. Discussion

Although meta-analyses on this topic have been conducted in general athletic populations [11,13], research focusing on para-athletes remains limited. This gap may be attributed, in part, to challenges associated with cold-water immersion for these athletes [3]. Therefore, the present study sought to assess the impact of various recovery methods—specifically, passive recovery (RP) and cold-water immersion (CWI)—on muscle damage in Paralympic powerlifting athletes. To enhance clarity, we have organized this discussion into two distinct sections (the effect on maximal isometric strength and biochemical markers of muscle damage).

### 4.1. Effect on Maximal Isometric Strength

CWI has been used to enhance post-training recovery [11]. However, contrary to this, CWI recovery after training would promote specific physiological adaptations and has shown negative effects on strength training [14]. This assertion is based on the detrimental effects of CWI on hypertrophy-related aspects, but it tends to improve indicators related to neural aspects, such as the power/force development rate [31,32]. Therefore, the application of CWI in post-exercise recovery could have negative effects on strength training aimed at morphological aspects and positive effects on rapid force generation, which is more focused on neural aspects [33].

Despite contradictory results in the literature regarding CWI after strength training [33,34,35], our study found a positive association between CWI recovery and strength indicators, consistent with other studies linking this method to post-exercise recovery, especially in terms of rapid strength recovery [36,37]. Given the highly specific nature of strength [3], strength training is subject to various interferences, including movement patterns, range and speed of motion, and load intensity, among others [2,4]. However, it is suggested that dynamic strength measures may interfere with strength gain assessments, increasing the likelihood that impairment was caused by CWI [34]. This was not the case in our study, likely due to the extremely specific population of Paralympic athletes evaluated.

### 4.2. Biochemical Markers of Muscle Damage

In this study, no differences were observed in the evaluated markers (TNF-α, IL-10), which is consistent with other studies [38,39], where CWI had no effect on plasma markers when assessed up to 72 h post-intervention. However, when evaluating IL-6, differences were observed in the CWI method between the time points of before, 15 min after, and 48 h after the recovery protocol. There were no differences between the methods, and no differences were observed in the passive recovery method.

When investigating the adaptations and consequences of training, it has been observed that exercise can induce muscle damage [2,4]. Muscle damage tends to trigger inflammatory responses aimed at muscle repair [7]. During recovery, pro-inflammatory cytokines tend to be released, such as TNF-α, which play a role in muscle injury and tissue degradation signaling [40]. Furthermore, the release of neutrophils, inflammatory cytokines (IL-6, among others), and reactive oxygen species tends to promote contractile dysfunction and increased catabolic activity, among other effects [41]. TNF-α, in particular, has been associated with a decrease in contractile capacity following exercise-induced damage [11]. However, our study did not show any significant difference between the passive and CWI recovery methods, and there was no significant increase in TNF-α in either method.

Contrary to these findings, regarding TNF-α, CWI recovery alone did not reduce the immune response to intense exercise [8]. Other studies have reported a slight increase or no increase in circulating TNF-α in different exercise protocols [42,43,44]. However, in research involving untrained subjects, TNF-α increased after eccentric exercises [8]. On the other hand, it has been reported that trained athletes exhibit a relatively higher TNF-α response, which was not observed in our study. Additionally, in line with our findings, it was reported that CWI recovery had no effect on TNF-α after exercise [8,45]. Our findings suggest that CWI recovery may not be effective regarding TNF-α.

CWI would act mainly through cold-induced vasoconstriction, which reduces blood flow to peripheral tissues, decreasing inflammation and post-exercise muscle edema [46]. CWI may help reduce delayed onset muscle soreness and provide faster recovery of muscle strength. Thus, the mechanisms by which CWI acts in relation to recovery would be through the reduction of blood lactate levels, where exposure to cold increases the rate of lactate removal, potentially accelerating metabolic recovery after intense exercise [47]. CWI would also modulate the inflammatory response, reducing the release of pro-inflammatory cytokines and limiting secondary muscle damage [46]. However, immersion in cold water would act in relation to protein synthesis, where there would be a reduction in inflammation that would be beneficial in the short term, but frequent exposure to cold after training would attenuate muscle adaptations in the long term, interfering in the anabolic processes necessary for muscle growth and strengthening [6,46,47]. It is important to note that interleukin-6 (IL-6) initially acts as a pro-inflammatory cytokine, being secreted by cells of the immune system in response to training, among others, which would stimulate the production of other cytokines and the acute inflammatory response [48]. However, at higher concentrations or in specific contexts, IL-6 can play an anti-inflammatory role, inhibiting the action of pro-inflammatory cytokines, such as TNF-alpha and IL-1, in addition to promoting the production of anti-inflammatory cytokines such as IL-10 [49]. This dual function of IL-6 is important for the regulation of the immune response and tissue recovery, both in inflammatory processes and in the body’s homeostasis.

Regarding cytokines, it was observed that muscle contraction resulting from exercise would produce muscle damage, with the release of the myokine IL-6 [50]. Furthermore, the increase in IL-6 levels after exercise tends to promote an increase in anti-inflammatory cytokines such as IL-10 [51]. Another study observed the association of IL-6 with an anti-inflammatory profile, resulting in a decrease in TNF-α and an increase in IL-10 levels [52]. Similarly, another study indicated that IL-6 has a modulatory effect on exercise and also has an anti-inflammatory effect [53], which is consistent with our findings. Thus, there appears to be an association between IL-6/IL-10, indicating an improvement in the inflammatory profile after high-intensity exercise [54].

Regarding IL-6, it tends to limit movement, acting as a protector against fatigue, particularly in prolonged or intense exercises [55]. On the other hand, IL-6 is believed to stimulate cellular apoptosis [56], potentially amplifying the inflammatory response [55,56]. This tends to trigger the activation and degradation of damaged cells, including muscle cells, through inflammatory signaling pathways [57]. Consequently, this can lead to a decrease in muscle function and strength loss [56], which plays a role in the subsequent recovery [58].

Another important point is that inflammatory events have a reparative effect and contribute to muscle remodeling in response to training [56]. In line with our findings, another study observed that IL-6 levels practically returned to their initial values after 24 h, a timing consistent with our study [59].

Our study has some limitations. One limitation is the sample size; despite the sample being comprised of national and international level athletes, the number of athletes who participated in the study was limited. However, there are only ten weight categories for males and, to consider high-level athletes, we considered the ratio of weight lifted to body weight, a value greater than 1.4, and that they should be being ranked among the top 10 in the country in their respective categories, which in itself limited the number of athletes. For future studies, we will try to associate with other national-level teams in order to increase the number of athletes. Our study did not monitor dietary intake during the intervention, since the aim of the study was to assess the effect of recovery on the athletes’ usual training routine, including diet and sleep, which were not monitored.

## 5. Conclusions

Muscle damage can result from metabolic, structural, and microvascular changes, leading to both systemic and local effects. In light of this, the results indicated that the interventions affect some aspects of local recovery, but systemic effects still persist. Furthermore, the results suggest that the CWI method outperformed passive recovery at the 24 h mark. CWI was better in terms of static strength indicators, with the MIF being observed to remain more stable with CWI when compared to PR. At 24 h, CWI showed better performance than PR in terms of the MIF, time to MIF, and RFD compared to the post-training moment with PR, with CWI still showing better values than PR at this same moment. Moreover, CWI at 48 h was superior, particularly in terms of time to MIF. Based on the findings, CWI can be considered an important method that could lead to better recovery compared to passive methods, especially concerning static strength indicators at the 24 and 48 h marks after training. Practical applications for our findings would be the sporadic use of cold water immersion as a way to facilitate the recovery process of Paralympic powerlifting athletes, either after a more intense training session, or even aiming at a better recovery in relation to a specific action, such as a competition, aiding in recovery and consequently improving the response to training or performance. However, in view of practical applications, cold water immersion was observed as a procedure that improved muscle pain and provided better neuromuscular recovery compared to other therapies, mainly involving the use of water at normal temperature. In this sense, the use of cold-water immersion (CWI) has proven effective in reducing exercise-induced muscle damage (EIMD) [60], being indicated not only for Paralympic powerlifting athletes, but for athletes from other modalities who worked with higher loads and more intense training.

## Figures and Tables

**Table 1:** Characterization of subjects.

Variables	Values
Age (years)	27.08 ± 4.66
Body Weight (kg)	84.58 ± 18.48
Experience (years)	3.21 ± 0.53
1RM Bench Press Test (kg)	150.17 ± 27.46 *
1RM/Body Weight	1.80 ± 0.23 **

* All athletes with loads that keep them in the top 10 of their national categories. ** Bench press values above 1.4 would be considered elite athletes [21].

**Table 2:** Static strength indicators (mean ± SD; 95% CI) at various time points concerning passive recovery and cold-water immersion in Paralympic powerlifting training.

	MIF	Time to MIF	RFD
PR Before (a) X ± SD (IC95%)	**877.06 ± 292.32 c** **(680.68–1073.45)**	**1.90 ± 1.03 e** **(1.21–2.59)**	**2431.64 ± 779.30 e** **(1908.10–2955.18)**
CWI Before (b) X ± SD (IC95%)	846.70 ± 241.62 (684.38–1009.02)	1.90 ± 0.73 (1.41–2.39)	2349.66 ± 649.15 (1913.56–2785.77)
PR 15 min (c) X ± SD (IC95%)	**664.79 ± 169.58 g** **(550.87–778.71)**	**3.94 ± 1.44 a,d** **(2.98–4.90)**	**1851.78 ± 521.89 a** **(1501.18–2202.39)**
CWI 15 min (d) X ± SD (IC95%)	671.57 ± 111.02 (596.99–746.16)	3.40 ± 1.73 (2.24–4.56)	1865.60 ± 385.52 (1606.60–2124.60)
PR 24 h (e) X ± SD (IC95%)	**655.78 ± 151.47 a** **(554.02–757.54)**	**2.96 ± 0.55 f** **(2.59–3.33)**	**1891.06 ± 453.32 f** **(1586.51–2195.60)**
CWI 24 h (f) X ± SD (IC95%)	**799.88 ± 132.43 e** **(710.92–888.85)**	2.61 ± 0.61 (2.20–3.03)	2229.24 ± 370.29 (1980.48–2478.01)
PR 48 h (g) X ± SD (IC95%)	**792.33 ± 215.00 c** **(647.89–936.77)**	**2.19 ± 1.54 c** **(1.16–3.23)**	2203.62 ± 594.03 (1804.55–2602.70)
CWI 48 h (h) (X ± SD) (IC95%)	907,81 ± 255,55 (736.12–1079.49)	1.74 ± 1.37 d (0.81–2.66)	2500.08 ± 681.56 (2042.20–2957.95)
*p*	a = 0.046 * c = 0.011 * e = 0.011 # g = 0.048 *	a = 0.021 * c = 0.012 * d = 0.007 # e = 0.012 * f < 0.001 # g = 0.037 #	a = 0.040 * e = 0.031 * f = 0.024 #
F	* = 5.364 # = 3.906	* = 9.914 # = 8.578	* = 5.334 # = 3.205
η^2^p	0.349 * 0.281 #	0.498 * 0.462 #	0.348 * 0.243 #

*p* ≤ 0.05 (two way ANOVA and Bonferroni post hoc test). In the statistical analysis, the letters from “a” to “h” indicate which conditions and at what time points differences were observed. * Inter-Class; # Intra-Class. (η^2^p): small effect (≤0.05), moderate effect (0.05 to 0.25), large effect (0.25 to 0.50), and very large effect (>0.50). PR: passive recovery; CWI: cold-water immersion; Before: pre-Test; 15 min: after 15 min; 2 h: after 2 h; 24 h: after 24 h; 48 h: after 48 h. NS: No significant differences. The letters that appear before the *p* values refer to the types of recovery and the moments, where we have the letter “a” representing PR before, “b” CWI before, and so on. The letters in the Initial column, from "a" to "h" refer to the moments (before, after, 24 h and 48 h later) and the type of recovery (PR and CWI). The results in bold in the MIF, Time and RFD columns, and the letters after the mean and standard deviation values refer to the moment and corresponding methods of the first column.

## Data Availability

The data that support this study can be obtained at https://www.ufs.br, accessed on 11 January 2024, or the data will be made available on request.
